# Ablation of ventricular tachycardia after septal myectomy for hypertrophic cardiomyopathy

**DOI:** 10.1002/joa3.13022

**Published:** 2024-03-12

**Authors:** Abdullah Orhan Demirtas, Sheldon M. Singh

**Affiliations:** ^1^ Department of Cardiology Adana City Training and Research Hospital Adana Turkey; ^2^ Sunnybrook Health Sciences Centre, Department of Medicine, Faculty of Medicine University of Toronto Toronto Ontario Canada

**Keywords:** catheter ablation, hypertrophic cardiomyopathy, implantable cardioverter implantation, septal myectomy, ventricular tachycardia

## Abstract

Electrocardiography and 3D mapping images of the case.

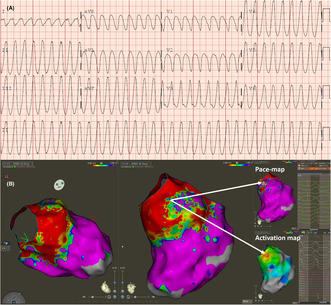

Managing symptoms and preventing sudden death are core elements when managing hypertrophic cardiomyopathy (HCM) patients. Surgical septal myectomy can achieve both of these goals.^1^ While there is some suggestion that surgical myectomy may offer some protection against ventricular arrhythmia, one study of postseptal myectomy patients with implantable defibrillators suggested a 4%/year rate of appropriate defibrillator shocks.^2^ Mechanisms of ventricular arrhythmia postseptal myectomy have not been reported. We report a case of monomorphic ventricular tachycardia (VT) in a patient with HCM a decade postseptal myectomy. Our case provides insight into one possible mechanism of ventricular arrhythmia postseptal myectomy.

A 61‐year‐old male patient underwent a septal myectomy 10 years prior. Because of the absence of risk factors for sudden death an implantable defibrillator was not offered postmyectomy. His baseline ECG was atrial fibrillation with a left bundle block, a known finding postseptal myectomy. The patient was admitted to the hospital because of recurrent syncopal episodes. He was found to be in incessant ventricular tachycardia (VT; Figure 1A). Echocardiography demonstrated that the basal septum was thin (0.75 cm) consistent with the prior myectomy. The remainder of the left ventricle (LV) was moderately hypertrophic without a LV outflow tract gradient. The patient's LV ejection fraction, which was previously normal, was reported to be reduced at 45%. The reduction in the LV function was presumed to be owing to the incessant nature of the ventricular arrhythmia. The patient was triaged for a VT ablation procedure because of ongoing arrhythmia refractory to the use of amiodarone and lidocaine.

**FIGURE 1:**
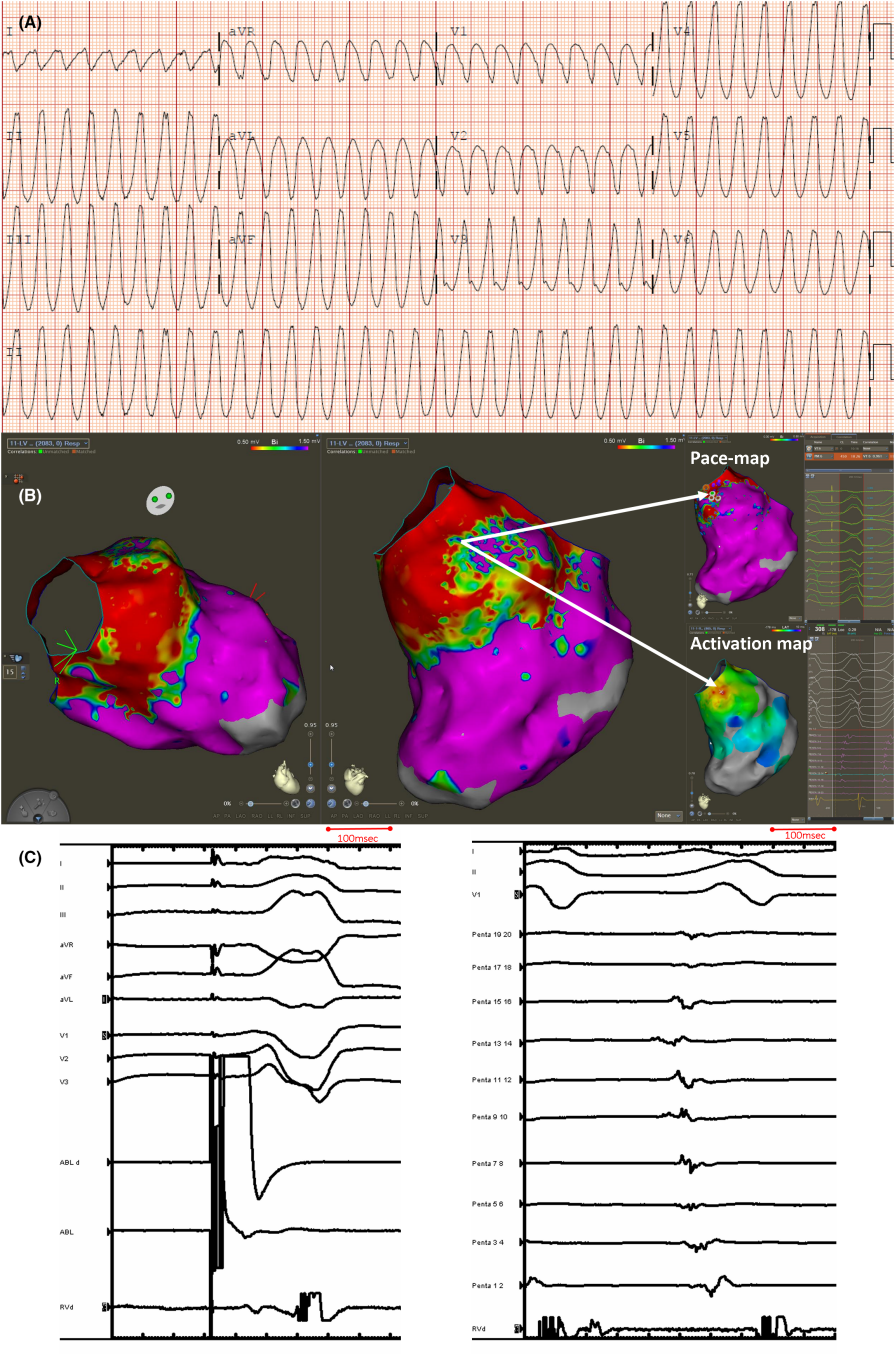
(A) Twelve lead of ventricular tachycardia. (B) Scarring noted in the basal septal LV region (red color corresponds to voltage <0.5 mV which is dense scar; whereas colors that are pink correspond to normal voltage >1.5 mV). The area with patchy scar in left ventricle anterior outflow tract area was noted to be earliest during VT and also had the pace map site. (C) Local electrogram during pacing at the site of successful ablation demonstrating a low amplitude fractionated late signal (left). Electrograms at the earliest site during ventricular tachycardia (right).

Mapping of the right ventricular outflow tract, left ventricle, aortic root and the distal coronary sinus including the proximal 1–2 cm of the anterior interventricular vein was undertaken using the CARTO mapping system, Pentaray multielectrode mapping catheter and the Thermocool SmartTouch Surround Flow ablation catheter (Biosense Webster). Endocardial biventricular voltage maps were created during right ventricular pacing and demonstrated low voltage areas (<1.5 mV) localized to the basal LV septum and adjacent peri‐aortic region (Figure 1B). This area was corresponded to the location of the surgical intervention. Patchy scar adjacent to the dense scar region related to the myectomy was noted.

There was a paucity of late potentials. Pace mapping in the region of patchy scar adjacent to the septal myectomy demonstrated a 96% pacematch to the clinical VT with a short stimulus to QRS suggesting pacing at the exit site for the VT. A local activation timing map was created during VT and demonstrated the earliest region with focal breakout in the same region. The earliest local signal was approximately 40 ms prior to the QRS in VT. Signals throughout the entire diastolic period were not present during LV endocardial mapping and only 60% of the VT cycle length was obtained with LV endocardial mapping (Figure 1C). The findings suggested either a focal tachycardia or exit of a VT circuit deeper within the septal myocardium. Entrainment was not performed prior to ablation to confirm whether the VT mechanism was reentrant or focal in nature. Ablation at this site promptly terminated VT and rendered it noninducible. Additional ablation lesions (40 W for 2 min in duration) were performed in this region in an attempt to homogenize the scar.

The patient subsequently underwent implantable cardioverter implantation. He has been followed for 1‐year and remains free of ventricular arrhythmia off anti‐arrhythmic drugs. His LV systolic function is now normal.

We report a case of VT presenting late after septal myectomy in a patient without ongoing traditional risk factors for sudden death. Our report highlights that fibrosis and scaring in the region of and adjacent to the myectomy site may serve as a source for focal or reentrant arrhythmia. The delay from surgery to VT in this case suggests that progressive fibrosis or alteration in this tissue may be required to facilitate arrhythmia. As demonstrated in this case, and supported by the literature, catheter ablation in HCM patients may have a high degree of success.^3^

## CONFLICT OF INTEREST STATEMENT

Authors declare no conflict of interests for this article.

## PATIENT CONSENT STATEMENT

Available.
